# Variation in the Abundance of *OsHAK1* Transcript Underlies the Differential Salinity Tolerance of an *indica* and a *japonica* Rice Cultivar

**DOI:** 10.3389/fpls.2017.02216

**Published:** 2018-01-05

**Authors:** Guang Chen, Chaolei Liu, Zhenyu Gao, Yu Zhang, Anpeng Zhang, Li Zhu, Jiang Hu, Deyong Ren, Ling Yu, Guohua Xu, Qian Qian

**Affiliations:** ^1^State Key Laboratory of Rice Biology, China National Rice Research Institute, Hangzhou, China; ^2^State Key Laboratory of Crop Genetics and Germplasm Enhancement, MOA Key Laboratory of Plant Nutrition and Fertilization in Lower-Middle Reaches of the Yangtze River, Nanjing Agricultural University, Nanjing, China

**Keywords:** salinity stress, *OsHAK1*, Na^+^ and K^+^ homeostasis, *Oryza sativa*, 9311, Nipponbare

## Abstract

Salinity imposes a major constraint over the productivity of rice. A set of chromosome segment substitution lines (CSSLs), derived from a cross between the *japonica* type cultivar (cv.) Nipponbare (salinity sensitive) and the *indica* type cv. 9311 (moderately tolerant), was scored using a hydroponics system for their salinity tolerance at the seedling stage. Two of the CSSLs, which share a ∼1.2 Mbp stretch of chromosome 4 derived from cv. Nipponbare, were as sensitive to the stress as cv. Nipponbare itself. Fine mapping based on an F_2_ population bred from a backcross between one of these CSSLs and cv. 9311 narrowed this region to 95 Kbp, within which only one gene (*OsHAK1*) exhibited a differential (lower) transcript abundance in cv. Nipponbare and the two CSSLs compared to in cv. 9311. The gene was up-regulated by exposure to salinity stress both in the root and the shoot, while a knockout mutant proved to be more salinity sensitive than its wild type with respect to its growth at both the vegetative and reproductive stages. Seedlings over-expressing *OsHAK1* were more tolerant than wild type, displaying a superior photosynthetic rate, a higher leaf chlorophyll content, an enhanced accumulation of proline and a reduced level of lipid peroxidation. At the transcriptome level, the over-expression of *OsHAK1* stimulated a number of stress-responsive genes as well as four genes known to be involved in Na^+^ homeostasis and the salinity response (*OsHKT1;5*, *OsSOS1*, *OsLti6a* and *OsLti6b*). When the stress was applied at booting through to maturity, the *OsHAK1* over-expressors out-yielded wild type by 25%, and no negative pleiotropic effects were expressed in plants gown under non-saline conditions. The level of expression of *OsHAK1* was correlated with Na^+^/K^+^ homeostasis, which implies that the gene should be explored a target for molecular approaches to the improvement of salinity tolerance in rice.

## Introduction

Salinity imposes a serious constraint over crop productivity, especially for crops grown under irrigation or in coastal lowlands prone to seawater ingress (Campbell et al., 2017). Over 800 Mha of arable land is affected by salinity to some degree, an area which represents some 6% of the total used for cropping (Munns and Tester, 2008). While certain cereal species show a substantial level of tolerance to salinity, rice does not (Munns and Tester, 2008). Its sensitivity depends on the developmental stage of the plant (Fageria, 1985; Khan et al., 1997); it is particularly vulnerable during its early vegetative growth, so that stand density is compromised in salinity-affected fields (Lutts et al., 1995; Gregorio et al., 2002). Some cultivars exhibit their highest sensitivity at the time of tillering and panicle initiation (Zeng et al., 2002).

Surveys of rice germplasm have suggested the presence of considerable genetic variation for the ability to withstand salinity (Negrão et al., 2011): the most tolerant entries known are originate from coastal environments (Flowers and Yeo, 1995; Gregorio et al., 2002; Flowers, 2004; Thomson et al., 2010). The genetic control of salinity tolerance, revealed using the quantitative trait locus approach (Koyama et al., 2001; Lin et al., 2004; Thomson et al., 2010; Ahmadi et al., 2011; Negrão et al., 2011; Platten et al., 2013), involves genes present in a number of genomic regions. The best characterized of these forms part of the short arm of chromosome 1, the site of *HKT1;5* (Lin et al., 2004; Thomson et al., 2010; Ahmadi et al., 2011), a gene which has been shown to encode a sodium ion (Na^+^) transporter which regulates shoot Na^+^/K^+^ homeostasis during an episode of salinity stress (Ren et al., 2005).

The potassium cation (K^+^) is involved in many aspects of plant growth and development (Ahmad et al., 2016). In saline soils, its uptake across the plasma membrane is impeded by competition with Na^+^, resulting in a fall in the plant tissue’s [K^+^]/[Na^+^] ratio (Schachtman and Liu, 1999). Plants have evolved a number of means of both promoting the uptake and transport of K^+^ and limiting the accumulation of Na^+^ in sensitive tissues (Schachtman and Liu, 1999; Luan et al., 2009; Cui et al., 2016). The transporters encoded by *KT/HAK/KUP* genes are thought to be key for maintaining an acceptable [K^+^]/[Na^+^] ratio, and hence represent an important component of plant tolerance to salinity stress (Chen et al., 2015b). A number of *KT/HAK/KUP* genes have been shown to be inducible by salinity stress (Maathuis, 2006). Both *HvHAK1* (Santa-María et al., 1997) and *PhaHAK1* (Takahashi et al., 2007) are likely involved in low affinity Na^+^ influx. A single altered residue in the HAK5 protein produced by several plant species has the effect of raising both the protein’s affinity for K^+^ and the level of salinity tolerance exhibited by yeast cells expressing this protein heterologously (Alemán et al., 2014). The rice OsHAK2 protein was sensitive to extracellular Na^+^ and transports Na^+^ more effectively than K^+^ (Horie et al., 2011b). The expression of *OsHAK5* in tobacco BY2 cells enhances their salinity tolerance via a boost in their capacity to accumulate K^+^ without affecting Na^+^ uptake (Horie et al., 2011b). The over-expression of *OsHAK5* in rice increases the shoot [K^+^]/[Na^+^] ratio and enhances the level of its salinity stress tolerance (Yang et al., 2014), while the knocking out of *OsHAK1* retards growth and depresses K^+^ accumulation (Chen et al., 2015b).

Here, a set of rice chromosome segment substitution lines (CSSLs), developed from a cross between the cultivars Nipponbare and 9311, was used to characterize variation for salinity tolerance at the seedling stage and to identify the genetic basis of K^+^ and Na^+^ homeostasis. The main outcome of the experiments was a demonstration that the level of *OsHAK1* expression has an impact on the plant’s ability to tolerate salinity, suggesting the gene could be targeted in breeding programs seeking to boost the salinity tolerance of rice.

## Materials and Methods

### Plant Materials and Growing Conditions

From a new set of CSSLs developed from the cross between the *japonica* type cv. Nipponbare (donor) and the *indica* type cv. 9311 (recipient), the two lines CSSL-1 and CSSL-2 were chosen to analyze the genetic basis of the differential salinity response of cv. 9311 and cv. Nipponbare. Both CSSLs were poor performers when challenged by salinity stress. To create a mapping population, CSSL-1 was back-crossed with cv. 9311 and the F_1_ plants allowed to self-pollinate. The origin and properties of two transgenic lines, one harboring *pOsHAK1::GUS* and the other *pOsHAK1::OsHAK1*, along with the *oshak1* mutant in a background of either cv. Dongjin or cv. Manan, have been described by Chen et al. (2015b). To raise seedlings under hydroponics, the grains were surface-sterilized and allowed to germinate in a nutrient-containing solution (Li et al., 2006); after 1 week, uniformly sized seedlings were transferred to a hydroponics system and exposed to a 14 h photoperiod, under a temperature regime of 30°C/22°C and a relative humidity level of ∼70%. The nutrient solution was replaced every 2 days. After a further week, the solution was salinized by the addition of 100 mM NaCl, to which the seedlings were exposed for a week. The harvested material was immersed in 0.1 mM CaSO_4_ for 5 min, then separated into root and shoot, which were weighed separately and assayed for their [Na^+^] (Na^+^ content) and [K^+^], following methods given by Chen et al. (2018). For a soil-based experiment set up to compare the performance of the *OsHAK1* transgenic and wild type (WT) plants, single plants were grown in 10 kg of air-dried loam for 5 weeks, then exposed to a 3 week period of salinity stress (1.5 g NaCl per kg of soil) by following the Zeng et al. (2017) procedure; a duplicate set of plants, to which no salt was provided, was grown as a control. A second experiment applied the same level of stress to plants once they had reached the booting stage, with the stress maintained through to plant maturity. Each treatment was replicated five times.

### Quantitative Real Time PCR (qRT-PCR)

The procedures used to conduct qRT-PCR followed those specified by Chen et al. (2015a). RNA was extracted from root and shoot tissue of both WT and transgenic, hydroponically grown seedlings, and from the youngest two leaves of soil-grown plants. The rice *Ubq* gene (LOC_Os03g13170) was chosen as the reference sequence, and relative transcript abundances were calculated following the suggestion of Li Y. et al. (2014). The stress-responsive genes assayed were *OsP5CS1* (pyrroline-5-carboxylate synthetase; Sripinyowanich et al., 2013), *OsDREB2A* (dehydration-responsive element-binding protein; Dubouzet et al., 2003), *OsAP37* (adaptor protein; Oh et al., 2009), and *OsERD1* (early responsive to dehydration; a homolog of *Arabidopsis thaliana AtERD1*; Kiyosue et al., 1994), while the transporter genes were *OsHKT1;5* (Na^+^ transporter; Ren et al., 2005), *OsSOS1* (Na^+^/H^+^ antiporter; Martínez-Atienza et al., 2007), *OsLti6a* and *OsLti6b* (proteins involved in maintaining the integrity of the plasma membrane during abiotic stress; Mekawy et al., 2015). The sequences of the various primers used are listed in Supplementary Table 1.

### Quantification of GUS Activity

GUS activity was measured in transgenic plants harboring the *pOsHAK1::GUS* construct as described by Chen et al. (2015a).

### Functional Complementation of OsHAK1 in Yeast

The coding sequence of *OsHAK1* was inserted into pYES2 (Invitrogen, Carlsbad, CA, United States) driven by the inducible Gal1 promoter, then transformed into the salinity-sensitive yeast strain AXT3 [Δena1::HIS3::ena4,nha1::LEU2,Δnhx1::TRP1] (Li Q. et al., 2014). The cells were grown for 3 days on solidified AP medium at 30°C (Horie et al., 2011a). Their ion content was measured in three independent clones per construct, using the Xu et al. (2008) method, with the following modifications: the cells were cultured at 30°C in 2 L liquid AP medium either with or without 50 mM NaCl with shaking (200 rpm). When the OD_600_ reached 0.8, they were harvested by centrifugation (5 min, 8000 rpm) and rinsed twice in liquid AP medium with no added K^+^ or Na^+^. They were then dried at 70°C for 72 h, weighed and digested overnight in 1 mL HNO_3_. After diluting with de-ionized water, the content of both K^+^ and Na^+^ was measured using an Optima 2100DV ICP-emission spectrometer (PerkinElmer, Waltham, MA, United States).

### Quantification of Electrolyte Leakage

The methods used to quantify electrolyte leakage were as described by Chen et al. (2017).

### Measurement of Photosynthetic Rate

Seedling rates of photosynthesis were measured between 9.00 and 11.00 am using a Li-COR6400 portable photosynthesis system equipped with a LED leaf cuvette (Li-COR, Lincoln, NE, United States), as described by Li et al. (2016). At least five individuals per genotype per each stress treatment were assayed.

### Determination of Leaf Chlorophyll Content

Following the procedures given by Li et al. (2016), leaves of WT and transgenic plants were weighed and extracted in aqueous 95% (v/v) ethanol. The absorbance of the extract was recorded at both 663 nm and 645 nm using a UV2400 spectrophotometer (Shimadzu, Kyoto, Japan). Chlorophyll concentrations (mg per g leaf fresh weight) were derived from the expression 8.02A_663_ + 20.21A_645_.

### Leaf Hydrogen Peroxide (H_2_O_2_), Malondialdehyde (MDA) and Proline Content

The H_2_O_2_ content of leaf samples was determined following their equilibration in 5.3 mM TiCl_4_ dissolved in 3.7 M H_2_SO_4_, based on the Mostofa and Fujita (2013) procedure. Leaf MDA contents were assessed following Chen et al. (2017), and leaf proline contents following Bates et al. (1973).

### Statistical Analysis

Analyses of variance were carried out using routines implemented in SPSS v10 software (SPSS Inc., Chicago, IL, United States). Statistically significant (*P* < 0.05) differences between the performance either of the various genotypes and WT or between different treatments (as determined by Tukey’s test), are denoted by the inclusion on the histograms of a different letter or an asterisk.

## Results

### The Relative Salinity Tolerance of the CSSLs and Their Parental Cultivars

**FIGURE 1:**
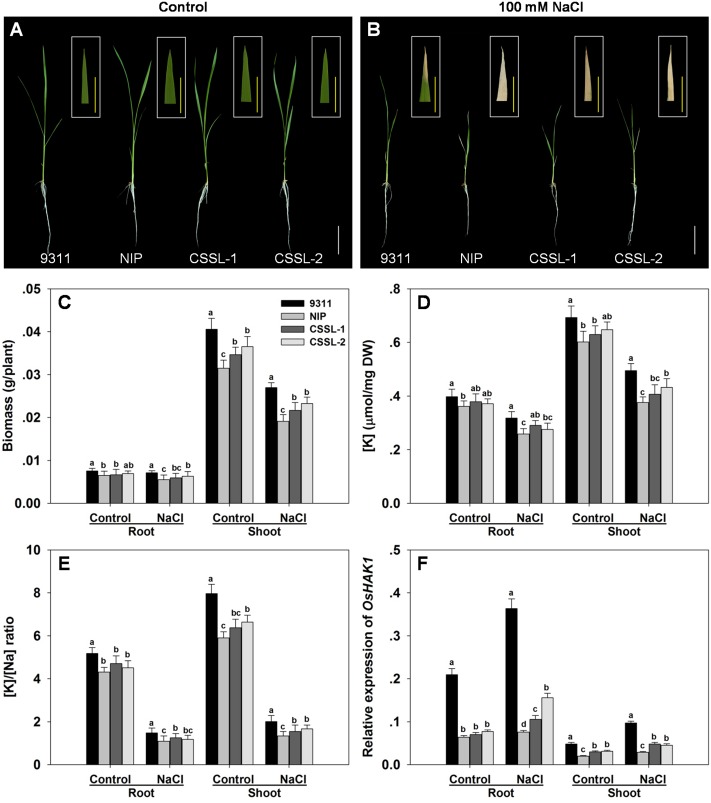
The contrasting salinity tolerance of the rice cvs Nipponbare and 9311, and two derived CSSLs. Seedlings were exposed to 100 mM NaCl for 7 days. **(A,B)** The growth and appearance of the plants raised in **(A)** the absence of NaCl, **(B)** the presence of 100 mM NaCl. White bars: 5 cm, yellow bars: 1 cm. **(C)** Dry matter accumulation, **(D)** [K^+^], **(E)** [K^+^]/[Na^+^] ratio and **(F)** the abundance of *OsHAK1* transcript. Values shown in the form of mean ± SE (*n* = 5). Means labeled with the same lower case letter did not differ significantly from one another (*P* < 0.05).

At the seedling stage, cv. 9311 was more tolerant of a 7 days exposure to 100 mM NaCl than was cv. Nipponbare. The cv. Nipponbare seedlings displayed both extensive wilting and foliar chlorosis, whereas the cv. 9311 ones became only mildly wilted, displaying withering just at the tip of their leaf blades (**Figures 1A,B**). Under control conditions, cv. 9311 seedlings accumulated the most root and shoot dry matter, followed by those of CSSL-1, CSSL-2 and cv. Nipponbare. The effect of the salinity stress was to reduce the dry matter accumulated by cv. 9311 by 29%, that by CSSL-1 by 34%, that by CSSL-2 by 31% and that by cv. Nipponbare by 36% (**Figure 1C**). Measurement of the root and shoot [K^+^] and [Na^+^] in seedlings grown under salinity stress showed that the parental cultivars differed significantly from one another: [K^+^] was 10% greater in the root and 15% greater in the shoot of stressed cv. 9311 seedlings than of cv. Nipponbare seedlings (**Figure 1D**). The effect of the stress on the two CSSLs was to raise the root and shoot [K^+^] above the level induced in the cv. Nipponbare seedlings, but below that induced in cv. 9311 seedlings. The root and shoot [K^+^]/[Na^+^] ratios behaved in a similar fashion (**Figure 1E**).

**FIGURE 2:**
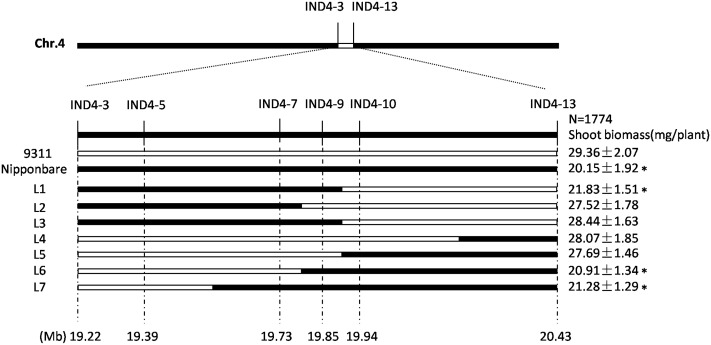
Graphical genotypes uncovered in the critical region of chromosome 4 among F_2_ progeny derived from the cross CSSL-1 x cv. 9311. The unfilled regions represent those homozygous for cv. 9311 alleles, and the filled ones represent those homozygous for cv. Nipponbare alleles. Mean shoot biomass values ± SE (*n* = 5) are shown on the right. ^∗^Mean differs significantly (*P* < 0.05) between cv. 9311 and genotypes carrying the critical chromosome 4 segment inherited from cv. Nipponbare.

The CSSL-1 and CSSL-2 genomes harbor a non-identical spectrum of cv. Nipponbare segments, but they share an overlap on chromosome 4 defined by the two InDel markers IND4-3 and IND4-13. The 1,774 F_2_ segregants derived from the cross CSSL-1 × cv. 9311 were tested with respect to their seedling response to salinity and also genotyped with respect to IND4-3 and IND4-13, along with a further four newly developed InDel markers lying within the chromosome 4 segment (Supplementary Table 2). Combining the genotypic and phenotypic data reduced the genomic window harboring gene(s) involved in the differential salinity response of cvs Nipponbare and 9311 to a 95 Kbp segment bounded by IND4-9 and IND4-10 (**Figure 2**), a region which, according to the Rice Genome Annotation Project^1^, contains 11 annotated genes. One of these genes is *OsHAK1*, which encodes a high affinity potassium transporter important for the regulation of K^+^ homeostasis (Supplementary Table 3). When the transcription in both the root and shoot of all eleven annotated genes was assessed, it was clear that the abundance of *OsHAK1* transcript was different between cv. Nipponbare and both CSSLs compared to cv. 9311 (**Figure 1F**), while there was no evidence for differential transcription among any of the other ten genes (data not shown). A sequence analysis of the entire *OsHAK1* coding sequence revealed a single synonymous nucleotide substitution between cvs Nipponbare and 9311 (Supplementary Figure 1).

### The Response of *OsHAK1* to Salinity Stress

**FIGURE 3:**
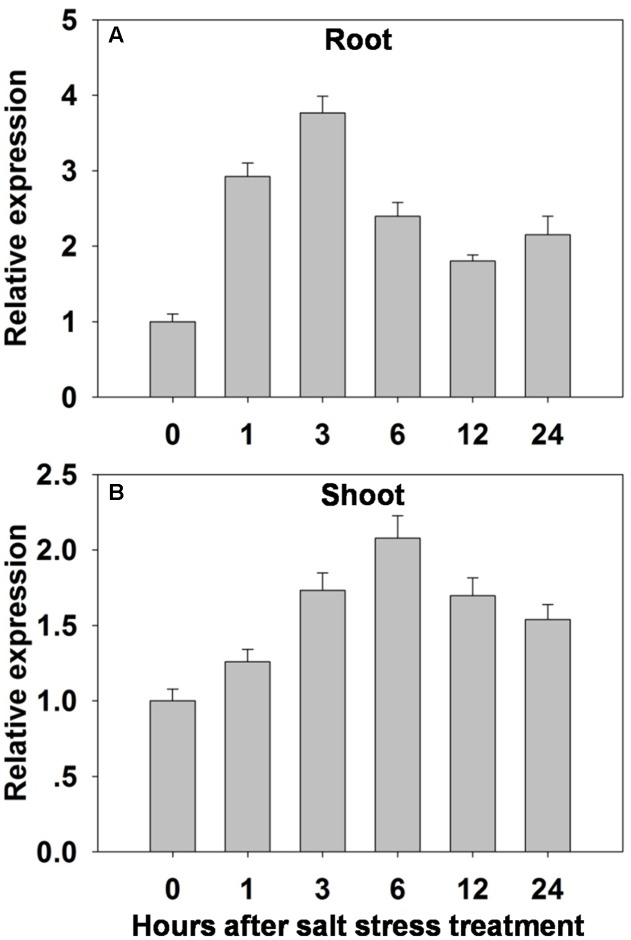
The effect of salinity stress (100 mM NaCl for 0–24 h) on the transcription of *OsHAK1* in **(A)** the roots and **(B)** the shoots of WT seedlings. Transcript abundances were derived using qRT-PCR, with *Ubq* chosen as the reference sequence. The abundance of *OsHAK1* transcript prior to the salinity treatment was set arbitrarily as 1. The whiskers indicate the SE (*n* = 3).

A qRT-PCR analysis showed that *OsHAK1* was induced in both root and shoot – but particularly in the former - by exposure of the seedlings to 100 mM NaCl (**Figure 3**). In the roots, the abundance of transcript rose within 1 h of the imposition of the stress, peaked to a level equivalent to three times the background within 3 h, then gradually declined over the rest of the assessment period (**Figure 3A**). In the shoots, the abundance of transcript was highest after 6 h of exposure (double the background level) (**Figure 3B**). *OsHAK1* expression was also examined in the roots and leaf blades of plants by analyzing GUS activity in plants carrying the *pOsHAK1::GUS* transgene (Supplementary Figures 2A–D). The transgene’s activity was significantly boosted by exposure to 100 mM NaCl, and more so in the root than in the shoot (Supplementary Figure 2E), consistent with the transcriptomic analysis (**Figure 3**).

### The Functionality of *OsHAK1* in a Yeast Mutant Lacking Na^+^/H^+^ Antiporter Activity

**FIGURE 4:**
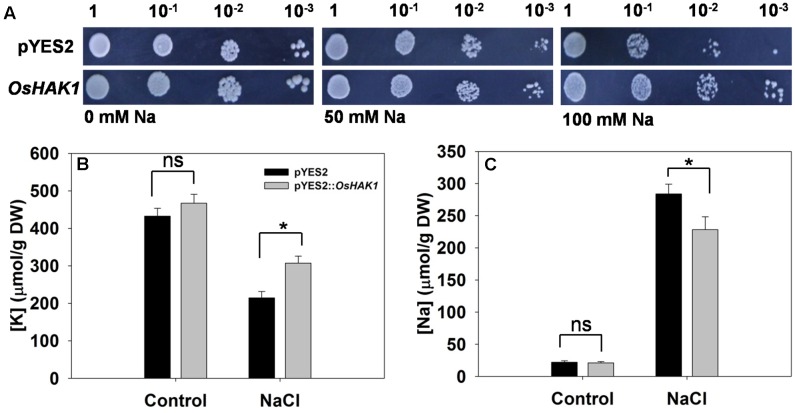
Complementation of a salinity-sensitive yeast mutant by *OsHAK1*. **(A)** Yeast cells harboring an empty vector (pYES2) are shown in the upper row, and those harboring *OsHAK1* in the lower row. The **(B)** [K^+^] and **(C)** [Na^+^] of yeast cells grown either in the absence (control) or presence of 50 mM NaCl. Data represent the mean ± SE (*n* = 3). Significant differences (*P* < 0.05) between the performance of cells harboring the empty vector and those harboring the *OsHAK1* transgene are indicated by an asterisk. ns, non-significant difference; DW, dry weight.

Cells of yeast strain AXT3 transformed with *OsHAK1* grew better on AP medium containing either 50 mM or 100 mM NaCl than did those carrying just an empty pYES2 vector (**Figure 4A**). Measurement of their [K^+^] and [Na^+^] showed that in the absence of salinity stress, the control and transgenic cells were indistinguishable (**Figures 4B,C**). Exposure to the stress raised [Na^+^] and decreased [K^+^] in both control and transgenic cells. However, [Na^+^] in the *OsHAK1* expressors was markedly lower than in the non-expressors (**Figure 4C**), while [K^+^] was 43% greater (**Figure 4B**).

### The Effect on Seedling Growth of Altering the Level of *OsHAK1* Transcript

**FIGURE 5:**
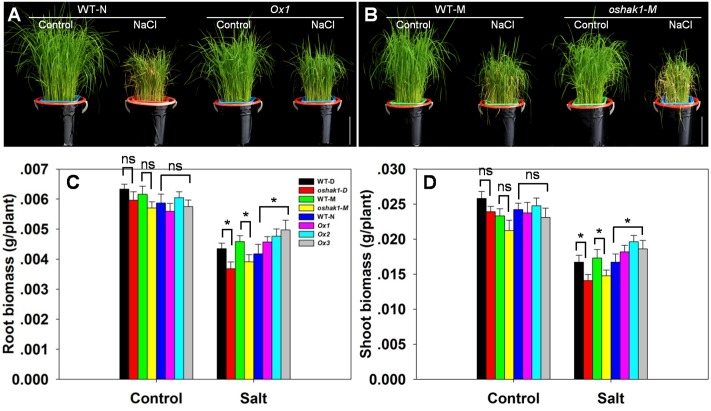
The effect of *OsHAK1* on the growth of rice seedlings. **(A)** The growth of an *OsHAK1* over-expressor (Ox1) and WT in either non-salinized (control) or salinized (100 mM NaCl) medium. **(B)** The growth of an *OsHAK1* knock-out mutant (*oshak1-M*) and its WT in either non-salinized (control) or salinized (100 mM NaCl) medium. Bar: 10 cm. **(C,D)** Dry matter accumulation of seedings raised in either non-salinized (control) or salinized (100 mM NaCl) medium: **(C)** the root and **(D)** the shoot. Values shown in the form mean ± SE (*n* = 5). Significant differences (*P* < 0.05) between the test genotype and WT are indicated by an asterisk. ns, non-significant difference; WT-D, cv. Dongjin; WT-N, cv. Nipponbare; WT-M, cv. Manan, *oshak1-D, oshak1-M*: *OsHAK1* knockout mutants in, respectively a cv. Dongjin and a cv. Manan background.

The response of hydroponically grown seedlings to exposure to 100 mM NaCl was compared between WT, *oshak1* knock-out mutant and *OsHAK1* over-expressing plants. No differences were detected when the plants were grown in the absence of salinity (**Figures 5A,B**), but the imposition of stress induced wilting and foliar chlorosis in WT and *oshak1*, but not in the *OsHAK1* over-expressor (**Figures 5A,B**). The stress did suppress shoot and root growth for all three genotypes, but the extent of the reduction was significantly less for the *OsHAK1* over-expressor than for the knock-out mutant plants. When challenged by salinity, the plant height, root dry matter and shoot dry matter attained by the over-expressor seedlings were all significantly greater than those attained by WT seedlings. The total dry matter accumulated by the knock-out mutants was about 85% of that accumulated by their respective WT (**Figures 5C,D**).

### The Effect of *OsHAK1* on Ion Uptake and the Transcription of Other Transporter Genes

**FIGURE 6:**
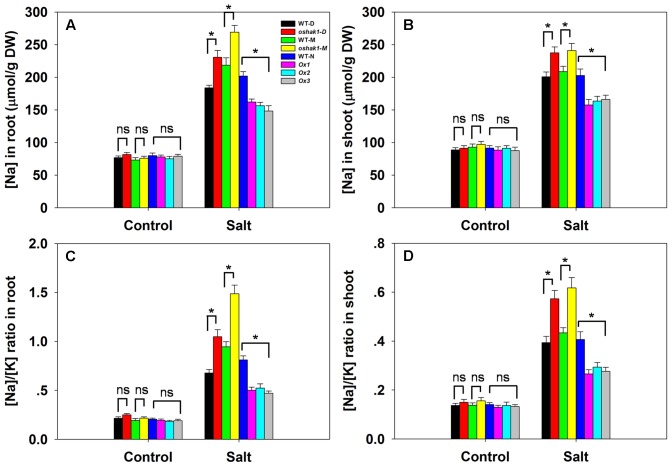
The effect of *OsHAK1* on Na^+^ accumulation and the [Na^+^]/[K^+^] ratio in seedlings challenged by salinity stress. [Na^+^] in **(A)** the root and **(B)** the shoot. The [Na^+^]/[K^+^] ratio in **(C)** the root and **(D)** the shoot. Values shown in the form mean ± SE (*n* = 5). Significant differences (*P* < 0.05) between the test genotype and WT are indicated by an asterisk. ns, non-significant difference. DW, dry weight.

**FIGURE 7:**
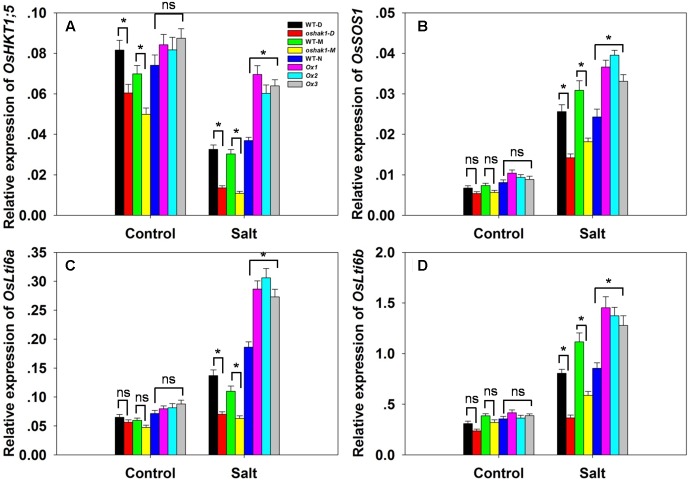
The abundance of transcript from genes encoding Na^+^ transporters. **(A–D)** The transcription in the roots of hydroponically-grown seedlings raised either in the presence or absence of salinity stress: **(A)** *OsHKT1;5*, **(B)** *OsSOS1*, **(C)** *OsLti6a*, **(D)** *OsLti6b.* Outputs derived from qRT-PCRs were normalized against the abundance of *Ubq* transcript. Values shown in the form mean ± SE (*n* = 3). Significant differences (*P* < 0.05) between the test genotype and WT are indicated by an asterisk. ns, non-significant difference.

There was no significant variation between the genotypes with respect to either [Na^+^] or the [Na^+^]/[K^+^] ratio in either root or shoot under control conditions; however, in the presence of 100 mM NaCl, compared to the performance of WT plants, the *oshak1* mutants over-accumulated Na^+^, while the *OsHAK1* over-expressors took up significantly less Na^+^ into both their roots and shoots (**Figures 6A,B**). The [Na^+^]/[K^+^] ratio was particularly high for the mutant seedlings, while it was maintained at a lower level in the over-expressors (**Figures 6C,D**). The qRT-PCR platform was deployed to quantify the transcription of four genes encoding a variety of transport proteins. *OsHKT1;5* was significantly down-regulated in the *oshak1* mutant plants under control conditions (**Figure 7A**). The presence of salinity down-regulated *OsHKT1;5* in each of the genotypes, although the extent of the reduction was less for the *OsHAK1* over-expressor than for the knock-out mutant plants (**Figure 7A**). For the other three genes, under control conditions, there was no variation in the level of their transcription between the various lines, but under salinity stress the genes were all up-regulated in the over-expressor relative to WT and down-regulated in the knock-out mutants (**Figures 7B,D**).

### The Effect of *OsHAK1* on K^+^ and Na^+^ Homeostasis at the Tillering Stage

When grown in soil, the *oshak1* mutants took up substantially more Na^+^ into both their roots and shoots under control conditions, but the differences in tissue [Na^+^] and [Na^+^]/[K^+^] ratio between the over-expressor and WT plants were not statistically significant (Supplementary Figure 3). In salinized soil, the over-expressor plants accumulated significantly less Na^+^ in both their shoot and root tissue than did WT plants (Supplementary Figures 3A,B). The [Na^+^]/[K^+^] ratio in both the root and shoot was notably lower in the over-expressor plants than in WT ones (Supplementary Figures 3C,D). In contrast, relative to WT plants, the knock-out mutants accumulated about 43% more Na^+^ in their shoot and about 54% more in their root tissue (Supplementary Figures 3A,B), resulting in a marked increase in the [Na^+^]/[K^+^] ratio (Supplementary Figures 3C,D).

### The Effect of Varying the Level of *OsHAK1* Expression on the Cellular Physiology of Salinity-Stressed Plants

**FIGURE 8:**
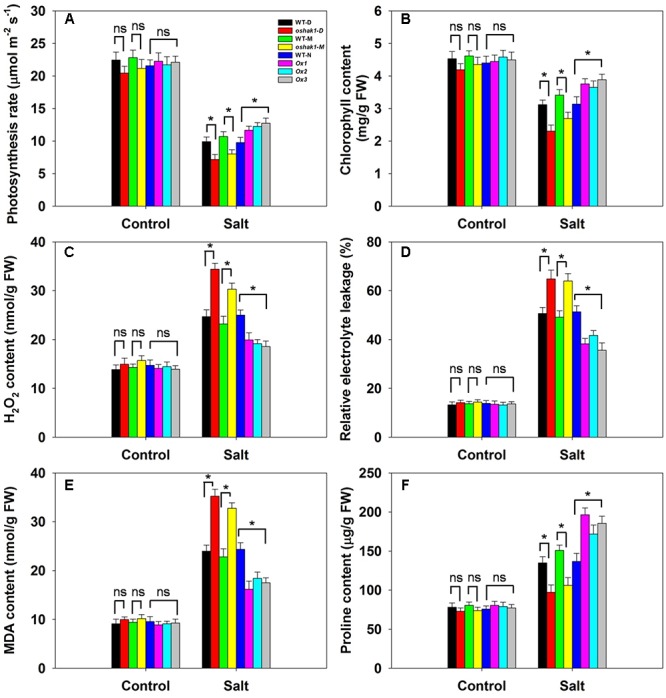
The effect of *OsHAK1* on the physiology of soil-grown plants exposed to salinity stress. **(A)** Photosynthetic rate, **(B)** leaf chlorophyll content, **(C)** H_2_O_2_ content, **(D)** relative electrolyte leakage, **(E)** MDA content and **(F)** proline content. Values shown in the form mean ± SE (*n* = 5). Significant differences (*P* < 0.05) between the test genotype and WT are indicated by an asterisk. ns, non-significant; FW, fresh weight.

Neither the photosynthetic rate nor the leaf chlorophyll content varied between the genotypes under control conditions (**Figures 8A,B**). The effect of salinity stress was to reduce the photosynthetic rate in all three genotypes, but the decline was more marked in WT than in *OsHAK1* over-expressor plants, resulting in the latter maintaining a significantly higher photosynthetic rate (**Figure 8A**). Similarly, the leaf chlorophyll content was reduced by the stress in each of the genotypes (**Figure 8B**). The *oshak1* mutants experienced the largest salinity stress-induced fall with respect to both photosynthetic rate and leaf chlorophyll content (**Figures 8A,B**). The *OsHAK1* over-expressor plants accumulated notably less H_2_O_2_ in response to salinity stress than did WT ones, while the H_2_O_2_ content of the knock-out mutant plants was ∼35% higher than that of their respective WT (**Figure 8C**). Under control conditions, there was no difference with respect to relative electrolyte leakage between the over-expressor and the WT plants, whereas after exposure to salinity, the over-expressor plants’ leaves suffered a significantly smaller decrease in relative electrolyte leakage than the WT plants’ leaves; the extent of leakage from the mutant plants’ leaves was substantially greater than that from their respective WT (**Figure 8D**). Similarly, the over-expression of *OsHAK1* reduced the accumulation of MDA: under salinity stress, their MDA content was 25% below that of WT plants, while the mutant plants accumulated 45% more than the WT (**Figure 8E**). When grown under control conditions, the proline content of the tissue of the over-expressor plants was similar to that of WT plants (**Figure 8F**). The effect of the imposed salinity stress was an increase in proline content in both the over-expressor and WT plants; even so, the over-expressor lines accumulated 35% more proline than the WT plants, whereas the knock-out mutant plants accumulated about 30% less proline than the WT plants (**Figure 8F**).

### Salinity Stressed Plants Expressing the *OsHAK1* Transgene Display an Altered Transcriptional Profile of Stress-Responsive Genes

**FIGURE 9:**
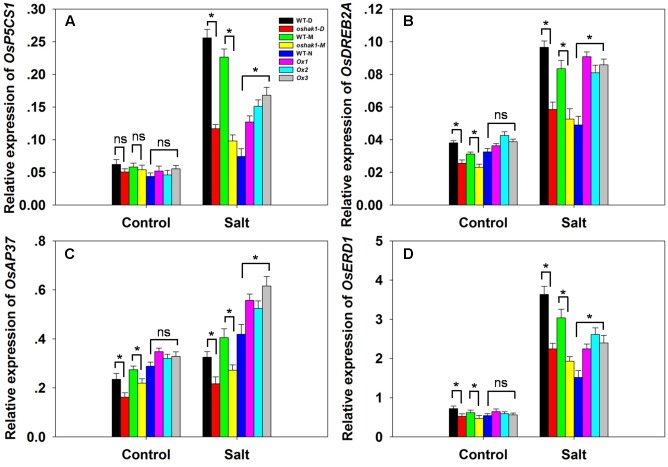
The effect of *OsHAK1* on the transcription of selected stress-responsive genes. **(A–D)** The transcription in the youngest two leaves of soil-grown plants in the presence or absence of salinity stress: **(A)** *OsP5CS1*, **(B)** *OsDREB2A*, **(C)** *OsAP37*, **(D)** *OsERD1*. Outputs derived from qRT-PCRs were normalized against the abundance of *Ubq* transcript. Values shown in the form mean ± SE (*n* = 3). Significant differences (*P* < 0.05) between the test genotype and WT are indicated by an asterisk. ns, non-significant difference.

The transcription of the stress-responsive genes *OsP5CS1*, *OsDREB2A*, *OsAP37* and *OsERD1* was monitored in WT, *OsHAK1* over-expressor and *oshak1* mutant plants. Under control conditions, the abundance of transcript did not vary between the WT and the over-expressor plants, but that of *OsDREB2A*, *OsAP37* and *OsERD1* was suppressed in the mutant plants (**Figures 9A–D**). In response to the salinity treatment, each of the four genes was notably up-regulated in the over-expressor plants. In the *oshak1* mutant plants, all four genes were less abundantly transcribed than in WT (**Figure 9**).

### The Over-Expression of *OsHAK1* Significantly Improved Salinity Tolerance at the Reproductive Stage

**FIGURE 10:**
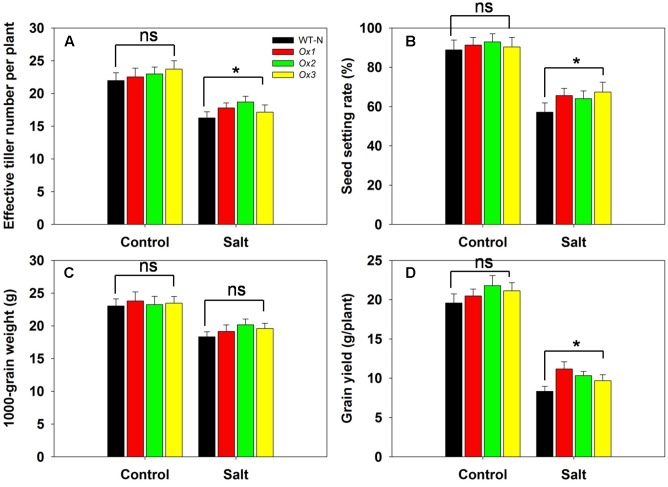
The over-expression of *OsHAK1* raises the level of rice’s salinity tolerance at the reproductive stage. The stress was applied at the booting stage and maintained through to maturity. **(A)** Effective tiller number per plant, **(B)** seed set, **(C)** 1000 grain weight and **(D)** grain yield per plant. Values shown in the form mean ± SE (*n* = 5). Significant differences (*P* < 0.05) between the over-expressor and WT are indicated by an asterisk. ns, non-significant difference.

*OsHAK1* over-expressor and WT plants were subjected to salinity stress at the booting stage through to maturity to assess the tolerance of the *OsHAK1* over-expressor when the stress was delayed until the switch from vegetative to reproductive growth. Under control conditions, there was little difference between the agronomic performance of the over-expressor and WT plants (**Figures 10A–D**). However, when salinity stress was imposed, the over-expressor plants produced 6–15% more effective tillers than the WT plants (**Figure 10A**), exhibited a 12–18% higher rate of spikelet fertility (**Figure 10B**), produced grain which were 5–10% heavier (**Figure 10C**) and yielded 17–35% more per plant (**Figure 10D**).

## Discussion

In rice, OsHAK1 maintains K^+^-mediated growth and is important for K^+^ acquisition and transport over a wide range of external [K^+^] (Chen et al., 2015b). As yet, its participation in the salinity stress response is unclear. A number of lines of evidence generated from the present experiments has revealed that OsHAK1 also makes a positive contribution to salinity tolerance. *OsHAK1* was more strongly transcribed in the more tolerant cv. 9311 than in cv. Nipponbare; it was inducible by salinity stress; changes in its transcript abundance correlated positively with tolerance at both the seedling and tillering stages, as well as with K^+^ and Na^+^ homeostasis and correlated negatively with lipid peroxidation; its transcription level affected the abundance of several stress-related genes, and finally its over-expression improved the productivity of plants challenged with salinity.

### The Up-regulation of *OsHAK1* Contributes to the Salinity Tolerance of cv. 9311

Considerable genetic variability for salinity tolerance has been identified in surveys of rice germplasm (Rahman M.A. et al., 2016). Both cv. Nipponbare and the two selected CSSLs proved to be more sensitive to salinity than was cv. 9311: their seedling root and shoot growth was inhibited more strongly, and their leaves suffered a greater degree of chlorosis (**Figures 1A,B**). A key component of salinity tolerance relates to Na^+^ and K^+^ homeostasis (Babu et al., 2017). The [K^+^] of salinity-stressed cv. 9311 root and shoot tissue was markedly higher than that of both cv. Nipponbare and the two selected CSSLs (**Figure 1D**), mirroring the observation made by Lee et al. (2003) that tolerant *indica* varieties feature a greater capacity to take up K^+^ and to limit the [Na^+^]/[K^+^] ratio in the shoot when plants are exposed to salinity stress. An adequate shoot and root cell [K^+^] is important for the maintenance of growth, as K^+^ is needed both to maintain the cell turgor needed to ensure cell expansion, and to act as a co-factor for numerous enzymes (Leigh and Wyn Jones, 1984). The two selected CSSLs differ genetically along chromosome 4; the critical difference was related by fine mapping to a 95 Kbp stretch, which harbors the gene *OsHAK1.* Of all of the 11 genes present in the segment, this was the only one which was differentially transcribed between cvs Nipponbare and 9311, especially when the plants were subjected to salinity stress (**Figure 1F**). The correlation between K^+^/Na^+^ homeostasis and the abundance of *OsHAK1* transcript suggests the possibility that *OsHAK1* is a key gene in the determination of salinity tolerance, as also proposed by Chen et al. (2015b).

### OsHAK1 Regulates K^+^ and Na^+^ Homeostasis to Enhance Salinity Stress Resistance

The ability of a plant to restrict the accumulation of Na^+^ is a central feature of salinity tolerance (Noreen et al., 2010; Cui et al., 2016). The *OsHAK1* over-expressors accumulated less Na^+^ and were thus able to maintain a lower [Na^+^]/[K^+^] ratio than could either WT or the *oshak1* knock-out mutant (**Figures 6A–D** and Supplementary Figures 3A–D). The greater accumulation of Na^+^ in the mutant probably reflected a combination of a higher rate of Na^+^ uptake by its roots, a higher rate of passive diffusion resulting from compromise to its cell membranes and a less efficient mechanism of sodium efflux (Islam et al., 2015). The over-expression of *OsHAK1* may therefore primarily enhance seedling growth under conditions of salinity stress by limiting the uptake of Na^+^ (**Figures 5**, **6A–D** and Supplementary Figures 3A–D). An ability to control the transport of Na^+^ from the root to the shoot is also considered to be a significant component of tolerance (Apel and Hirt, 2004; Foyer and Noctor, 2005; Munns and Tester, 2008; Cui et al., 2016). The *OsHAK1* over-expressors accumulated less Na^+^ in their shoot tissue (**Figures 5**, **6B**), thereby limiting the [Na^+^]/[K^+^] ratio (**Figures 6C,D**); this parameter is particularly critical for ensuring normal cell function under conditions of salinity stress (Su et al., 2015). The active removal of Na^+^ is thought to be controlled by the products of genes belonging to the *HKT* family (Hamamoto et al., 2015). For example, an RNAi-induced knock-down of the wheat gene *TaHKT1;5* results in an increased accumulation of Na^+^ in the leaves (Byrt et al., 2014). For this reason it was of interest to assay the expression of its rice homolog in the present materials: the indication was that the gene was down-regulated in the *oshak1* mutant, which can explain why the mutant accumulated more Na^+^ in its tissue than did the *OsHAK1* over-expressor plants (**Figures 5**, **6A,B**, **7A**).

PMP3 (plasma membrane protein 3) is a conserved hydrophobic protein expressed in response to a variety of abiotic stresses (Chidambaram et al., 2015). In yeast, the loss of PMP3 function promotes Na^+^ accumulation, enhances sensitivity to salinity and induces membrane hyperpolarization (Nylander et al., 2001). In both *A. thaliana* and *Avicennia marina*, raising the abundance of this protein has been associated with a reduction in shoot [Na^+^] and a partial alleviation of stress-induced growth inhibition (Mitsuya et al., 2006; Chidambaram et al., 2015). The rice genome harbors two genes encoding PMP3 homologs, namely *OsLti6a* and *OsLti6b.* When plants were exposed to salinity stress, both of these genes were transcribed at a lower level in the *oshak1* mutant than in WT (**Figures 7C,D**). According to Mekawy et al. (2015), *PMP3* gene products act to control the uptake of Na^+^ by the roots of a tolerant cultivar, thereby limiting its leaf [Na^+^]. The up-regulation of the two *OsLti6* genes in the *OsHAK1* over-expressors may be due to the induction of other Na^+^ transporters, since PMP3 proteins contribute indirectly to cation homeostasis by interacting with other ion transporters (Fu et al., 2012). The plasma membrane Na^+^/H^+^ exchanger encoded by *SOS1* is considered to be the major plant mediator of Na^+^ efflux (Kronzucker and Britto, 2011): it makes an important contribution to the maintenance of Na^+^ and K^+^ homeostasis (Yamaguchi et al., 2013) and is involved in the movement of Na^+^ through the xylem (Olías et al., 2009). Here, the up-regulation of *OsSOS1* in the *OsHAK1* over-expressor plants probably enhanced the plants’ ability to prevent the uptake of Na^+^, thereby lowering the [Na^+^]/[K^+^] ratio in their shoot (**Figures 6B**, **7B**).

### The Positive Effect of OsHAK1 on Root and Shoot Growth

The faster a shoot is able to grow, the greater the dilution effect on toxic ions such as Na^+^. Rapid growth in spite of the presence of salinity stress has been suggested to represent a reliable indicator of tolerance (Yeo and Flowers, 1986; Yeo et al., 1990; Rahman M.A. et al., 2016). Here, the over-expression of *OsHAK1* had a positive effect on the growth of both the root and the shoot, in contrast to the negative effect of its loss-of-function in the *oshak1* mutant (**Figure 5**). The boost to growth was not limited to seedlings, as soil-grown *OsHAK1* over-expressors stressed post booting also recorded an improved performance with respect to grain yield (**Figure 10**). The benefit of *OsHAK1* over-expression may have been due to its depressive effect on Na^+^ accumulation, in particular thereby supporting the uptake of K^+^. Alternatively (or additionally) it may have derived from its protection against chlorophyll loss (**Figures 8A,B**), and thereby its maintenance of a high photosynthetic rate.

### The Improved Salinity Tolerance Derived from *OsHAK1* Over-Expression Is Reflected in a Positive Effect on a Number of Traits

The over-expression of *OsHAK1* had a measurable effect on a number of physiological parameters: it lowered the cellular content of H_2_O_2_ (**Figure 8C**) and MDA (**Figure 8E**), and promoted that of proline (**Figure 8F**). A well-documented effect of salinity stress in rice is the accumulation of H_2_O_2_ (Mostofa et al., 2015; Rahman A. et al., 2016), so the over-expressor tissue likely experienced a lower level of oxidative damage. Cellular MDA content is commonly used as a surrogate for determining the extent of lipid peroxidation (Zhao et al., 2014); the conclusion that membrane damage was less severe in the *OsHAK1* over-expressor than in WT was supported by its reduced extent of electrolyte leakage (**Figure 8D**). Proline has long been considered to act as an osmoprotectant (Yancey et al., 1982), but its contribution to osmoregulaton in salinity-stressed rice is controversial (Lutts et al., 1996). Here, when exposed to salinity stress, the *OsHAK1* over-expressors accumulated significantly more proline than WT (**Figure 8F**). The product of the gene *P5CS1*, which is implicated in proline synthesis, has been identified by Zhu et al. (1998) as being a key component of abiotic stress tolerance. The gene can be induced by a variety of stress agents, resulting in an increased proline content, while its over-expression in rice has been shown to improve osmotolerance (Igarashi et al., 1997; Zhu et al., 1998). Here, the higher abundance of *OsP5CS1* transcript in tissue of the stressed *OsHAK1* over-expressors was fully consistent with the observed increase in their tissue proline content (**Figures 8F**, **9A**). It is possible that the extra proline could also have improved the plant’s capacity to retain water (Song et al., 2012). Proline has also been suggested to act as an antioxidant (Székely et al., 2008), so a higher proline content may have also contributed to limiting electrolyte leakage and lowering the MDA content (**Figures 8D–F**).

The qRT-PCR analysis revealed a correlation between the transcript abundance of certain stress-related genes and the expression of salinity tolerance in the *OsHAK1* over-expressors. The over-expression of a number of transcription factors has been shown to up-regulate various stress-related genes, an effect suggested to contribute toward an improved level of stress tolerance (Kasuga et al., 1999; Mukhopadhyay et al., 2004; Zhao et al., 2014; Hong et al., 2016; Jiang et al., 2016; Shen et al., 2017). Here, the transcription of four such stress-related genes was shown to be enhanced in salinity-challenged *OsHAK1* over-expressors (**Figure 9**). One of these was *P5CS1*, as mentioned previously. The second was *DREB2A*, a gene which encodes a transcription factor known to influence both salinity and dehydration stress tolerance (Cui et al., 2011; Mallikarjuna et al., 2011). The third was *AP37*, encoding a protein associated with tolerance to drought, salinity and low temperature at the vegetative stage and with grain yield under severe drought (Oh et al., 2009). The fourth was a homolog of the *A. thaliana* gene *AtERD1*, which is inducible by dehydration stress (Kiyosue et al., 1994); the *OsERD1* gene is significantly up-regulated in a rice plant over-expressing a gene encoding a NAC transcription factor (*ONAC022*), which displays an enhanced level of salinity tolerance (Hong et al., 2016). The suggestion is that the enhanced tolerance to salinity stress generated by *OsHAK1* over-expression is, at least in part, a consequence of the up-regulation of these genes. ABA signaling has long been implicated as an important trigger of the plant response to various abiotic agents, as it regulates a number of key stress-related genes (Zhao et al., 2014). The salinity induced up-regulation in the *OsHAK1* over-expressors of both early response genes such as *DREB2A* and *AP37*, and of late ones such as *P5CS1* (**Figure 9**) implies that the *OsHAK1* product participates in ABA signal transduction either directly or indirectly. As yet the mechanistic basis of this involvement is unclear.

## Conclusion

The present study has demonstrated that variation in the intensity of *OsHAK1* expression can explain a part of the difference in the salinity tolerance expressed by cvs 9311 and Nipponbare. When up-regulated, *OsHAK1* improves the tolerance of the rice plant to salinity stress by regulating K^+^ and Na^+^ homeostasis, by promoting the growth of the root and shoot, by favoring the accumulation of proline, by protecting against stress-induced damage to the plasma membranes and by activating a number of stress-related genes. The over-expression of many genes in rice, while potentially addressing a trait weakness (such as salinity tolerance), frequently has undesirable pleiotropic effects on development and/or productivity (Kasuga et al., 1999; Hsieh et al., 2002; Nakashima et al., 2007; Morran et al., 2011). The over-expression of *OsHAK1* did not, however, appear to induce any such effects on plants grown in the absence of salinity stress (**Figures 5**, **10**). Most promisingly, the over-expressor expressed its enhanced salinity tolerance under field conditions, recording a 25% increase in grain yield per plant compared to the WT (**Figure 10D**). The implication is that *OsHAK1* represents a favorable gene target for the breeding of rice cultivars able to maintain productivity in saline-affected soils. It would be of interest to test the effect of transferring the *OsHAK1* allele present in a salinity tolerant cultivar to a salinity sensitive one, in particular to determine whether such an allelic exchange would alter the K^+^ and Na^+^ homeostasis in a way which would enhance its salinity tolerance.

## Author Contributions

Conceived and designed the experiments: GC, GX, and QQ. Performed the experiments: GC, CL, YZ, and AZ. Analyzed the data: GC, CL, ZG, GX, and QQ. Contributed reagents/materials/analysis tools: LZ, JH, DR, and LY. Wrote and revised the paper: GC, ZG, GX, and QQ.

## Conflict of Interest Statement

The authors declare that the research was conducted in the absence of any commercial or financial relationships that could be construed as a potential conflict of interest.

## Footnotes

1 http://rice.plantbiology.msu.edu/cgi-bin/gbrowse/rice/
