# Supplementary material for: Variation in the Abundance of OsHAK1 Transcript Underlies the Differential Salinity Tolerance of an indica and a japonica Rice Cultivar
Source: Front Plant Sci. 2018 Jan 5;8:2216. doi: 10.3389/fpls.2017.02216 (PMC5760540; doi:10.3389/fpls.2017.02216)
Supplement: Supplementary file 1 [file Table_1.DOCX]

**Supplementary Table 1.** The sequence of primers used for qRT-PCR.

| Gene | Primer ID | Primer sequences | |
| --- | --- | --- | --- |
| *Ubq* | F(5’-3’) | | ACCCTGGCTGACTACAACATC |
|  | R(5’-3’) | | AGTTGACAGCCCTAGGGTG |
| *OsHAK1* | F(5’-3’) | | GTTGATGATGCTGATGTTGGAAG |
|  | R(5’-3’) | | CCAACACTTTCAGCTGAAAC |
| *OsHKT1;5* | F(5’-3’) | | CCCATCAACTACAGCGTCCT |
|  | R(5’-3’) | | AGCTGTACCCCGTGCTGA |
| *OsSOS1* | F(5’-3’) | | ATACTGAGTGGGGTTGTTATTGC |
|  | R(5’-3’) | | AAAGGTAAATTTCAAAAGGTACATGG |
| *OsLti6a* | F(5’-3’) | | CCTTCCAAGGTGATGGTGAA |
|  | R(5’-3’) | | CCGTCCAAAGAACCAGAAAA |
| *OsLti6b* | F(5’-3’) | | GCTCCAAACCGCTTCATCTA |
|  | R(5’-3’) | | CAAGAATTGGAGCACTCAGGA |
| *OsP5CS1* | F(5’-3’) | | GCTGACATGGATATGGCAAAAC |
|  | R(5’-3’) | | GTAAGGTCTCCATTGCATTGCA |
| *OsDREB2A* | F(5’-3’) | | GGCTGAGATCCGTGAACCAA |
|  | R(5’-3’) | | GGACCATACATTGCCCTTGC |
| *OsAP37* | F(5’-3’) | | TCCGATGTTTTGGTCCTCTG |
|  | R(5’-3’) | | TCCACGGTTTAGTCCATCTCATC |
| *OsERD1* | F(5’-3’) | | TCAAAGGGAAGACGAAGCATGG |
|  | R(5’-3’) | | GGGACGGAATACAACCATCTCA |
