# Supplementary material for: Variation in the Abundance of OsHAK1 Transcript Underlies the Differential Salinity Tolerance of an indica and a japonica Rice Cultivar
Source: Front Plant Sci. 2018 Jan 5;8:2216. doi: 10.3389/fpls.2017.02216 (PMC5760540; doi:10.3389/fpls.2017.02216)
Supplement: Supplementary file 2 [file Table_2.DOCX]

**Supplementary Table 2.** The sequence of primers used to assay for indels in the region of chromosome 4 targeted for fine mapping.

| Marker | Primer ID | Primer sequences | |  |
| --- | --- | --- | --- | --- |
| IND4-3 | F(5’-3’) | | TATTTTCGACTAAACTCGGC | |
|  | R(5’-3’) | | GACTACCCATCTGGACTCCT | |
| IND4-5 | F(5’-3’) | | ACTCCGACTCCGACTCCT | |
|  | R(5’-3’) | | AACAAAGAGGACGTGCGAT | |
| IND4-7 | F(5’-3’) | | TATGGGACAAAGGGAGTATG | |
|  | R(5’-3’) | | ATGTGTGAATAGGTGGGAAG | |
| IND4-9 | F(5’-3’) | | AAACCAATGGAACTTTTCCT | |
|  | R(5’-3’) | | TTCTTAGGGCTTATTTTGGA | |
| IND4-10 | F(5’-3’) | | TCCAGAGAAACAAATAGCTCA | |
|  | R(5’-3’) | | GCAAATTCTAAACACAAGGC | |
| IND4-13 | F(5’-3’) | | GTGGAGAGAAGGGAGAGAGT | |
|  | R(5’-3’) | | CAGACGAGAGAAAGATGGTG | |
