# Supplementary material for: Variation in the Abundance of OsHAK1 Transcript Underlies the Differential Salinity Tolerance of an indica and a japonica Rice Cultivar
Source: Front Plant Sci. 2018 Jan 5;8:2216. doi: 10.3389/fpls.2017.02216 (PMC5760540; doi:10.3389/fpls.2017.02216)
Supplement: Supplementary file 3 [file Table_3.DOCX]

**Supplementary Table 3.** Genes annotated in the key 95 Kbp genomic region of chromosome 4.

| Gene ID | Annotation |  |
| --- | --- | --- |
| LOC_Os04g32870 | pentatricopeptide repeat-containing protein, putative, expressed | |
| LOC_Os04g32880 | CBS domain containing membrane protein, putative, expressed | |
| LOC_Os04g32890 | retrotransposon protein, putative, unclassified, expressed | |
| LOC_Os04g32900 | transposon protein, putative, unclassified | |
| LOC_Os04g32910 | retrotransposon protein, putative, Ty1-copia subclass, expressed | |
| LOC_Os04g32920 | potassium transporter, putative, expressed | |
| LOC_Os04g32930 | DNA cross-link repair 1A protein, putative, expressed | |
| LOC_Os04g32940 | Leucine Rich Repeat family protein, expressed | |
| LOC_Os04g32950 | calreticulin precursor protein, putative, expressed | |
| LOC_Os04g32960 | TUDOR protein with multiple SNc domains, putative, expressed | |
| LOC_Os04g32970 | OTU-like cysteine protease family protein, putative, expressed | |
