# Supplementary material for: Variation in the Abundance of OsHAK1 Transcript Underlies the Differential Salinity Tolerance of an indica and a japonica Rice Cultivar
Source: Front Plant Sci. 2018 Jan 5;8:2216. doi: 10.3389/fpls.2017.02216 (PMC5760540; doi:10.3389/fpls.2017.02216)
Supplement: Supplementary file 4 [file Data_Sheet_1.PDF]

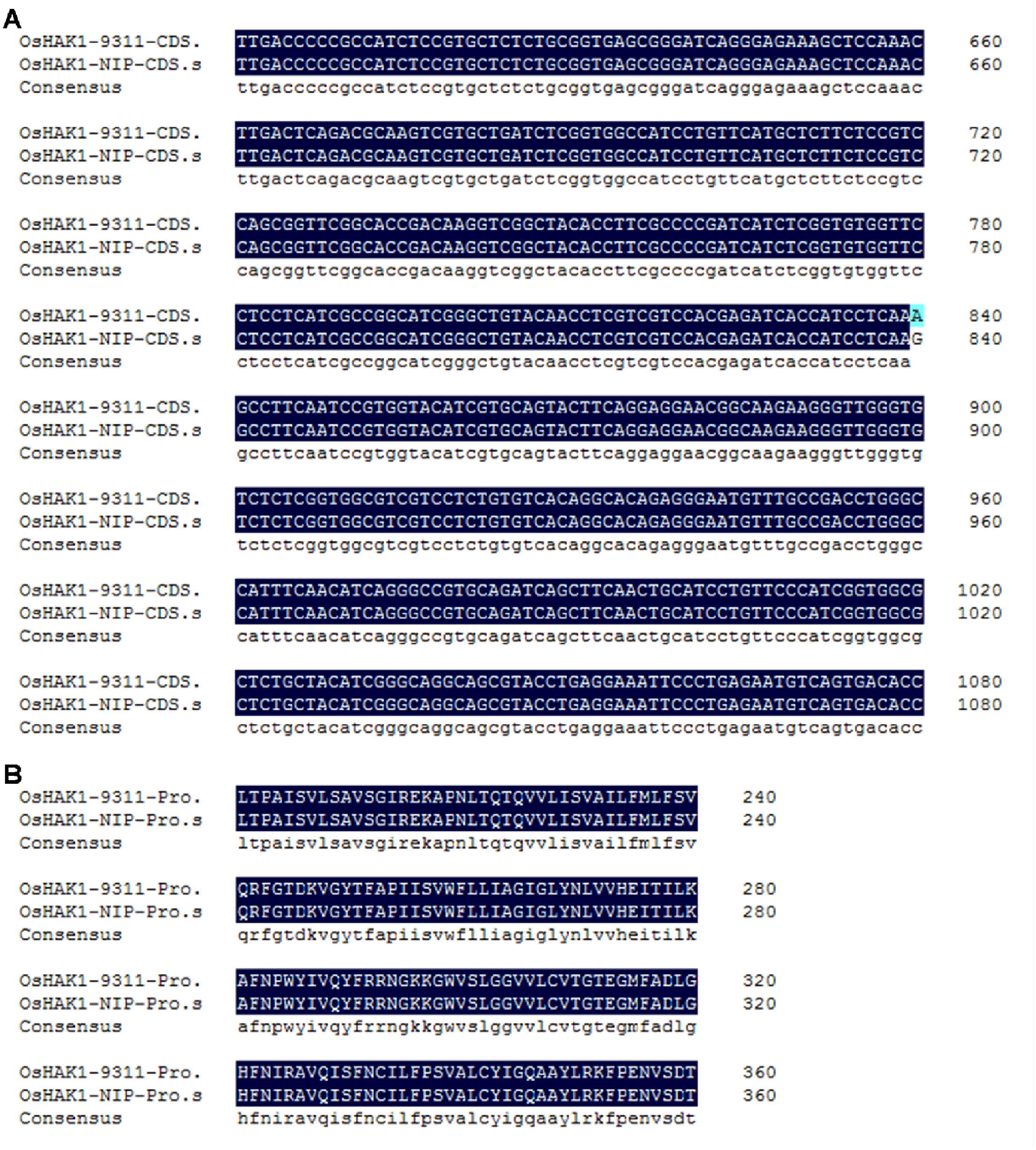

Supplementary Figure 1. A portion of the *OsHAK1* coding sequence and its predicted polypeptide product. (A) Nucleotide sequences, (B) polypeptide sequences of cvs Nipponbare and 9311. The numbers shown on the right indicate the position of nucleotides in (A), residues in (B).
