# Supplementary material for: Variation in the Abundance of OsHAK1 Transcript Underlies the Differential Salinity Tolerance of an indica and a japonica Rice Cultivar
Source: Front Plant Sci. 2018 Jan 5;8:2216. doi: 10.3389/fpls.2017.02216 (PMC5760540; doi:10.3389/fpls.2017.02216)
Supplement: Supplementary file 5 [file Data_Sheet_2.PDF]

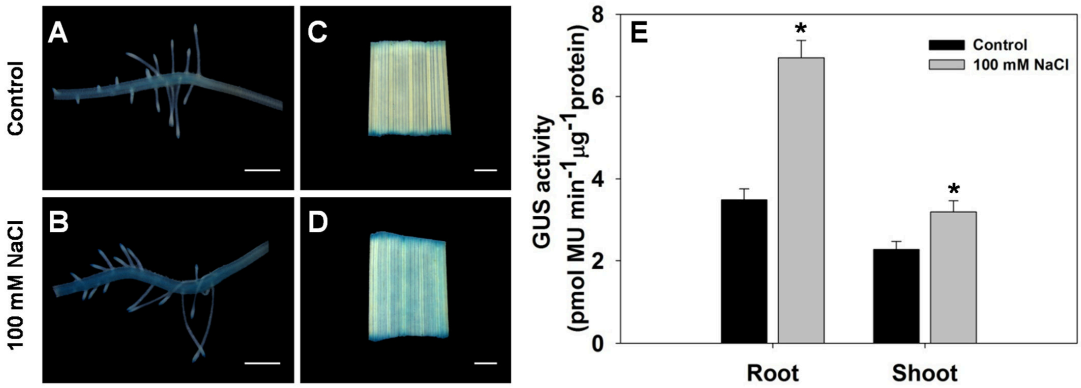

Supplementary Figure 2. The response of *OsHAK1* to salinity stress assayed in a plant harboring the construct *pOsHAK1::GUS*. Images of a GUS-stained (A,B) root and (C,D) leaf blade from seedlings raised under either control conditions (0 mM NaCl) or in the presence of 100 mM NaCl. Bar: 1 mm. (E) Quantification of GUS activity. Significant differences between treatment and control means indicated by an asterisk ($P<0.05$). The whiskers represent the SE ($n=3$).
