# Supplementary material for: Variation in the Abundance of OsHAK1 Transcript Underlies the Differential Salinity Tolerance of an indica and a japonica Rice Cultivar
Source: Front Plant Sci. 2018 Jan 5;8:2216. doi: 10.3389/fpls.2017.02216 (PMC5760540; doi:10.3389/fpls.2017.02216)
Supplement: Supplementary file 6 [file Data_Sheet_3.PDF]

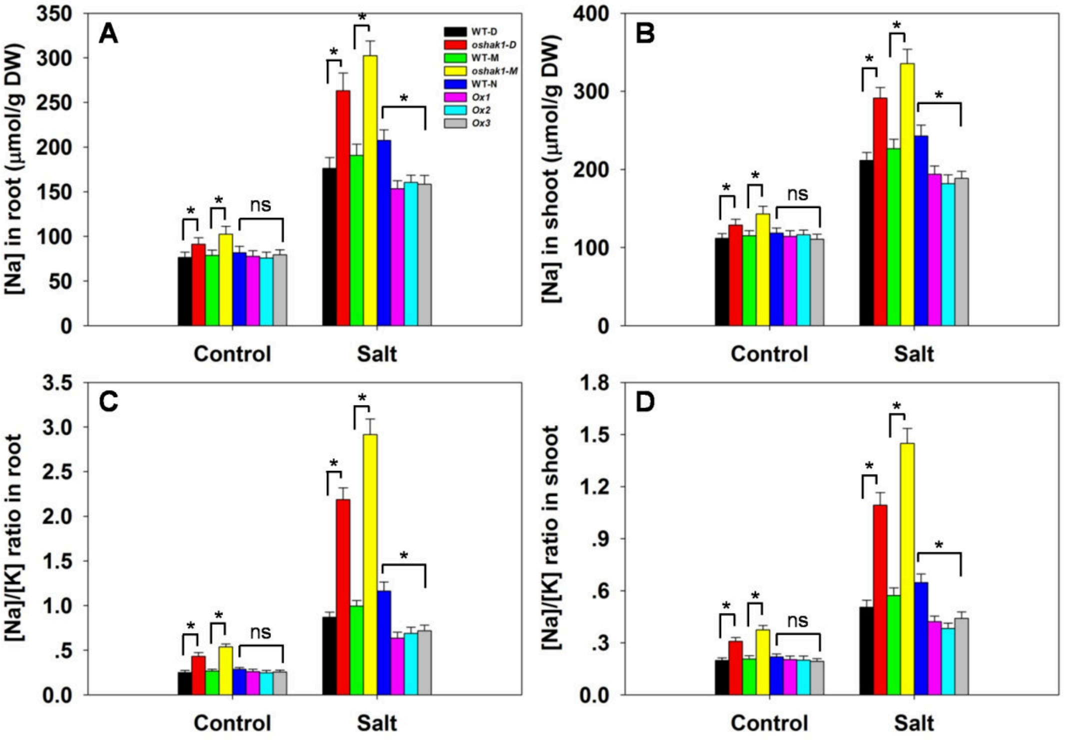

Supplementary Figure 3. The effect of *OsHAK1* on Na^+ accumulation and the $[Na^+]/[K^+]$ ratio in plants at the tillering stage challenged by salinity stress. $[Na^+]$ in (A) the root and (B) the shoot. The $[Na^+]/[K^+]$ ratio in (C) the root and (D) the shoot. Values shown in the form mean±SE (n=5). Significant differences (P<0.05) between the test genotype and WT are indicated by an asterisk. ns: non-significant difference; DW: dry weight.
